# Perceptions of readiness for interprofessional learning among Ethiopian medical residents at Addis Ababa University: a mixed methods study

**DOI:** 10.1186/s12909-024-05055-4

**Published:** 2024-01-25

**Authors:** Dereje Melka, Yonas Baheretibeb, Cynthia Whitehead

**Affiliations:** 1 Department of Neurology, Addis Ababa University School of Medicine, Addis Ababa, Ethiopia; 2 Department of Psychiatry, College of Health Sciences, Addis Ababa University, Addis Ababa, Ethiopia; 3 Department of Family and Community Medicine, University of Toronto, Director and Scientist at the Wilson Centre, Temerty Faculty of Medicine, University of Toronto and University Health Network, Toronto, Canada

**Keywords:** Interprofessional learning, Ethiopia, Perceptions of readiness of residents, Postgraduate

## Abstract

**Background:**

Interprofessional learning is an important approach to preparing residents for collaborative practice. Limited knowledge and readiness of residents for interprofessional learning is considered one of the barriers and challenges for applying Interprofessional learning. We aimed to assess the perceptions of readiness of medical residents for interprofessional learning in Ethiopia.

**Methods:**

We conducted a parallel mixed-methods study design to assess the perceptions of readiness for interprofessional learning among internal medicine and neurology residents of Tikur Anbessa Specialized Teaching Hospital in Addis Ababa, Ethiopia, from May 1 to June 30, 2021. One hundred one residents were included in the quantitative arm of the study, using the Readiness for Interprofessional Learning Scale (RIPLS) tool. All internal medicine and neurology residents who consented and were available during the study period were included. SPSS/PC version 25 software packages for statistical analysis (SPSS) was used for statistical analysis. Descriptive statistics were summarized as mean and standard deviation for continuous data as well as frequencies and percentages to describe categorical variables. Data were presented in tables. In addition, qualitative interviews were undertaken with six residents to further explore residents’ knowledge and readiness for IPL. Data were analyzed using a six-step thematic analysis.

**Results:**

Of the 101 residents surveyed, the majority of the study participants were male (74.3%). The total mean score of RIPLS was 96.7 ± 8.9. The teamwork and collaboration plus patient-centeredness sub-category of RIPLS got a higher score (total mean score: 59.3 ± 6.6 and 23.5 ± 2.5 respectively), whereas the professional identity sub-category got the lowest score (total mean score: 13.8 ± 4.7). Medical residents’ perceptions of readiness for interprofessional learning did not appear to be significantly influenced by their gender, age, year of professional experience before the postgraduate study, and department. Additionally, the qualitative interviews also revealed that interprofessional learning is generally understood as a relevant platform of learning by neurology and internal medicine residents.

**Conclusions:**

We found high scores on RIPLS for internal medicine and neurology postgraduate residents, and interprofessional learning is generally accepted as an appropriate platform for learning by the participants, which both suggest readiness for interprofessional learning. This may facilitate the implementation of interprofessional learning in the postgraduate medical curriculum in our setting. We recommend medical education developers in Ethiopia consider incorporating interprofessional learning models into future curriculum design.

**Supplementary Information:**

The online version contains supplementary material available at 10.1186/s12909-024-05055-4.

## Introduction

The increasing burden of chronic diseases and shortage of health care workers especially in low and middle income countries requires greater collaborative working between health professions [1]. The challenges of health care are increasingly complex and subject to frequent change. To meet these demands health professionals must work in collaboration with each other and the patient. To do this, students must learn how to work together with colleagues from different professions [2].

Interprofessional practice is becoming a new norm in healthcare, as health provider teams begin to shift away from the more traditional uni-disciplinary focus towards an improved collaborative team-oriented approach [3]. Delivery of comprehensive healthcare necessitates multidisciplinary teamwork [4]. There is growing interest in interprofessional learning (IPL) and in its education and research agenda to develop best practice models based on evidence of effectiveness [5].

IPL provides real-world practices and insights from several different health perspectives, leading to enriched consultation and discussion between diverse healthcare specialties [2]. Effective Interprofessional learning (IPL) can play a key role in preparing individual health professional students for future collaborative healthcare practice [6].

IPE is really still in its infancy in sub Saharan Africa (SSA), with more initiatives and evaluations needed to understand and measure its benefits on health care practice and patient outcomes [1].

The absence of interprofessional learning among professionals may negatively affect the quality of patient care. In collaborative processes, mutual respect and a match between the respective roles and expectations of different professionals enhance communication, cooperation, and contributions toward a common goal [7].

Interprofessional learning is often impacted by the perceptions of readiness of health professionals. To implement interprofessional learning approaches in the curriculum, it is important to understand the perceptions of readiness of students [8].

In comparison with lower resource settings, high resource settings over the past few decades have quickly developed interprofessional education programs and initiatives. Additionally, high resource settings have had greater capacity to expand interprofessional education than lower resource settings, such that interprofessional education initiatives differ across the world [9]. While education for collaboration is important across the health professions education continuum, to date, most of IPE programs have been implemented at the undergraduate level in both high resource and lower resource settings [9].

Out of 41 articles published between 1998 and 2021 in the review ‘The status and outcomes of interprofessional health education in sub-Saharan Africa: A systematic review’ the Democratic Republic of Congo, Ethiopia and Lesotho contributed only 7% of the presented IPE work [1]. To our knowledge, while there are several studies done in other parts of the world [7, 10–20] there have been few data published in English on the perceptions and readiness of medical residents’for interprofessional learning in Ethiopia. Our study objective, therefore, was to examine the perceptions of readiness of medical residents’ for interprofessional learning in the Internal Medicine and Neurology department, at Tikur Anbessa Specialized Teaching Hospital, Addis Ababa, Ethiopia.

## Methods

### Study design

We selected a parallel mixed-methods design, and compared and contrasted the findings from the quantitative and qualitative data, which were collected between May 1 to June 30, 2021. By triangulating the data, we aimed for a deeper understanding of the residents’ perceptions of readiness for IPL.

### Data collection tools, producers and sample size determination

For quantitative data, socio-demographic data, perceptions of readiness of residents on interprofessional learning data were collected using a structured questionnaire developed for this study. The paper-based structured questionnaire developed (which also include the RIPLS questionnaire) was administered. Readiness for Interprofessional Learning Scale (RIPLS) questionnaire was administered to examine the perceptions of readiness of residents for IPL. The RIPLS scale was selected since it is considered a valid tool for measuring the readiness of postgraduate healthcare professionals to engage in interprofessional learning in different parts of the world [21], although it has not specifically been validated in an Ethiopian or African context. The questionnaire was piloted on ten surgical residents; there were no clarity issues identified. All internal medicine and neurology residents in the year 2021, who gave informed consent, were included. The RIPLS questionnaire was self-administered to internal medicine and neurology residents in the study period just after their morning meeting session (during coffee break time). A trained data collector facilitated the data collection. 104 subjects were recruited for the study; three subjects were excluded due to the presence of incomplete data. The subjects used for the analysis were 101. The sample size planned was 119 subjects, which made the response rate of the study 87%.

### Study population and setting

Addis Ababa University is the only training center for neurologists in Ethiopia. Its main hospital is Tikur Anbessa specialized teaching hospital. Tikur Anbessa specialized teaching hospital is a tertiary teaching hospital with a total of 700–800 beds. The hospital is providing training (both undergraduate and postgraduate) in different major disciplines including Internal medicine, Gynecology/obstetrics, Pediatrics, Surgery, psychiatry, Emergency medicine, and Family medicine. It also provides subspecialty training. We specifically targeted residents from the internal medicine and neurology department considering that these groups of residents are spending their three years training altogether, the disciplines have extensive interprofessional interactions, and they are representative of residents who have multiple interprofessional interactions; which makes them an ideal group to study in terms of interprofessional learning. On average per year, there are 130–150 residents in both departments. Tikur Anbessa Specialized hospital was selected since it is the only teaching hospital providing neurology post-graduate training in the country currently. On the neurology and internal medicine residency training, IPL is not integrated in the curricula, even if informal interprofessional learning is occurring among health professionals in this setting.

The RIPLS has 23 self-reported items and a 5-point Likert Scale (Strongly agree = 5, agree = 4, neutral = 3, disagree = 2, and strongly disagree = 1) was used to analyze the residents’ responses. The tool has three different domains. Domain 1 focused on the aspects of teamwork and collaboration. Domain 2 focused on patient-centeredness. Domain 3 focused on the Sense of professional identity. A reversal coding was used for sense of professional identity subcategory elements. It is validated in different countries [14, 15, 21]. A higher score indicates greater readiness for Interprofessional learning on the "teamwork and collaboration” and "patient-centeredness" subscale and a lower score indicates greater readiness for interprofessional learning on the sense of professional identity.

For the qualitative data collection, we conducted semi-structured interviews to gain a deeper understanding of the answers provided in the questionnaires. The semi-structured interview guide was developed based on a literature review on interprofessional learning. The interview guide focused on main points regarding knowledge and readiness of residents on interprofesional learning. The semi structured interview guide was piloted on two surgery residents to check for the clarity of questions and there were no clarity issues. There were also no issues of clarity reported during data collection time. We chose residents from the Internal medicine and Neurology departments. At the end of the RIPLS questionnaire, all residents were asked if they were interested in volunteering to be interviewed. Their contact address was documented if they provided consent for that. Interview participants were selected randomly from both groups for interviews.

We used the concept of "information power" to guide an adequate sample size for our qualitative study. Information power indicates that the more information the sample holds, relevant to the actual study, the lower number of participants is needed. The size of a sample with sufficient information power depends on five areas: the aim of the study, sample specificity, use of established theory, quality of dialogue, and analysis strategy [22]. Based on the above analysis our study aim was narrow, the study participants held characteristics highly specific to the study aim, the study applies specific theoretical perspectives, and the quality of dialogue was strong since the interviewer has more than average background knowledge about the topic of the study (a public health professional having an academic level of assistant professor), and our analysis strategy was an in-depth analysis of narratives or dialogue details. Considering the above information power analysis for our study we needed 6 participants to get adequate information to address the study's aim.

Semi-structured interviews were conducted by a public health professional using the interview guide for a mean duration of 50 ± 5.4 min. The interviews were audio recorded and transcribed in preparation for data analysis. The interview question covered topics including residents’ knowledge of interprofessional education, issues of horizontal learning, issues of dynamics in the learning process, challenges in implementing interprofessional learning, and suggestions for successful implementation of IPE.

### Data analysis

For the quantitative data, the collected data were checked for completeness and entered into SPSS/PC version 25 software packages for statistical analysis (SPSS) for statistical analysis. Descriptive statistics were summarized as mean and standard deviation for continuous data as well as frequencies and percentages to describe categorical variables. We computed the total mean score and the mean score of each item for the RIPLS scale. Odds ratios and 95% confidence intervals were calculated. A *p* value less than 0.05 were considered a statistically significant association between assessed variables. For qualitative data, the researchers developed a group codebook to guide continued data analysis. The codebook was used to recode the interviews we used the concept of “information power”. All the interviews were audio-recorded. Interviews were transcribed from Amharic into English by the same data collector who conducted the interviews. The authors checked for the accuracy of the transcripts while taking care to preserve important local language concepts and nuances. A selection of the English translations was compared against the original Amharic transcripts by the PI and there was no significant difference noted. Data were analyzed using a six-step thematic analysis [23] (familiarization, coding, generating themes, reviewing themes, defining and naming themes, and writing up). Recordings were transcribed verbatim and read several times. Each interview was analyzed by dividing it into conversation sequences and examining them sentence by sentence. Researchers discussed naming subthemes linked to each category. The list of themes identified was commented on, reviewed, and refined to ensure internal consistency. Researchers removed overlaps across the various themes. The central themes were accurately described to identify their meanings. Memo-writing was used throughout data analysis to ensure the trustworthiness and rigor of the study.

### Ethical consideration

Protocol approval was obtained from the ethical review Committee of the Department of Health professional education and the Institutional Review Board (IRB) of the College of Health Sciences, Addis Ababa University. Study subjects provided informed written consent.

#### Theoretical framework

**Fig. 1:**
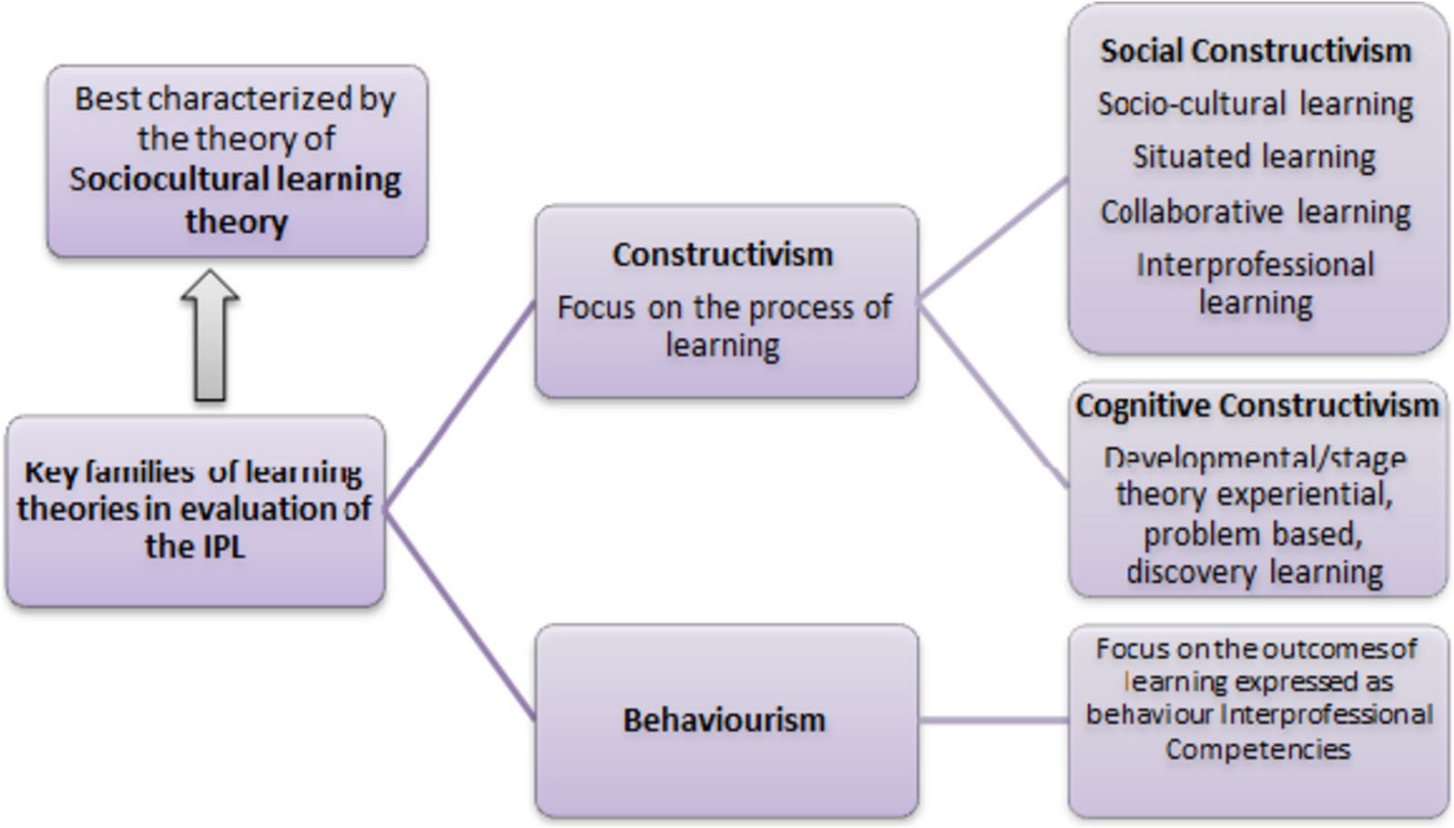
Theoretical framework of the study

In the evaluation of the IPL initiative, we draw upon constructivism as a learning theory that takes account of the process of learning. Whereas social constructivism views individual learning as being mediated by the environment; this approach is best characterized by the theory of socio-cultural learning theory. Considering the objective of this study was to determine knowledge and readiness for Interprofessional learning of medical residents, socio-cultural learning theory provides us with a theoretical framework that aligns with the study goal of assessing the perceptions of readiness of residents for interprofessional learning. Figure 1 situates socio-cultural learning theory within broader learning theories as a theoretical framework of the study [24].

## Results

### Sample characteristics

#### Quantitative data results

Seventy-five (74.3%) of the study subjects were male. The mean age of the study subjects was 28.5 ± 2.3 years. Table 1 shows the demographic of our study subjects.

**Table 1:** Frequency distributions of socio-demographic features of internal medicine and neurology residents, TASH, from May 1 – June 30, 2021

Variables	Number (%)
Gender	
Female	26(25.7)
Male	75(74.3)
Age	
Under28 years	58(57.4)
28—39 years	43(42.6)
Department	
Neurology	22(21.8)
Internal medicine	79(78.2)
Year of residency	
First-year	39(38.6)
Second year	30(29.7)
Third year	32(31.7)
Past professional experience before residency in months	
No experience	9(8.9)
6 – 36 months	74(73.3)
Above 36 months	18(17.8)
Previous exposure to the RIPILS questionnaire	
Yes	7(6.9)
No	94(93.1)

### Qualitative data results

The characteristics of the semi-structured interview subjects are presented in Table 2.

**Table 2:** The characteristics of the study subjects for semi-structured interview of internal medicine and neurology residents, TASH, from May 1 – June 30, 2021

No	Gender	Age	Department	Year of residency	Year of experience
1	Female	32	Neurology	3rd	3 years
2	Male	30	Internal medicine	3rd	8 months
3	Male	32	Neurology	^2^nd	3 years
4	Male	32	Neurology	3rd	3 years
5	Female	29	Neurology	2rd	3 years
6	Female	31	Internal medicine	3rd	3 years

### Residents’ perceptions of readiness for interprofessional learning

#### Quantitative data results

We found a high score of RIPLS on our postgraduate residents, which implies greater readiness for Interprofessional learning. The total mean score of RIPLS was 96.7 ± 8.9 and the minimum and maximum scores were 43 and 115 respectively. On the subcategories of the RIPLS score: teamwork and collaboration plus patient-centeredness scores were high (total mean score: 59.3 ± 6.6 and 23.5 ± 2.5 respectively),. However, on the professional identity sub-category of RIPLS the score was low (total mean score: 13.8 ± 4.7).

The highest mean score (4.80) was obtained for the statement “Patients ultimately benefit if health care professionals work together to solve patient problems” followed by the "Establishing trust with my patients is important to me” mean score (4.79). Residents obtained the lowest mean score, for the statement “I would feel uncomfortable if another health care professional knew more about a topic than I did (2.42)” and “There is little overlap between my role and that of other health care professionals (2.45)”. For the response on each items of RIPLS see the Supplementary material.

Medical Residents’ perceptions of readiness for Interprofessional learning did not appear to be significantly influenced by their gender, age, year of professional experience before the postgraduate study, and department Table 3.

**Table 3:** Results of interprofessional learning overall mean score with independent variables of neurology and internal medicine residents, TASH, from May 1 – June 30, 2021

Variables	Overall mean score of RIPLS (± SD)	Mean difference	95% CI	*P*-value
**Age**				
< 28	97.59 (± 7.7)	1.86087	-1.71707 – 5.43880	0.305
≥28	95.6 (± 10.4)			
**Sex**				
Male	96.9 (± 9.8)	0.95846	-3.10510 -5.02202	0.641
Female	95.9 (± 5.9)			
**Department**				
Neurology	96.7 (± 6.6)	0.01093	-4.29809 – 4.31996	0.996
Internal Medicine	96.6 (± 9.6)			
**Past professional experience before residency in months**				
No experience	93.6 (± 6.1)	-3.28228	-9.87246 – 3.30790	0.325
More than 6 months of experience	96.8 (± 9.7)			

#### Qualitative data results

To better understand the RIPLS results and to compare them with them, we conducted semi-structured interviews. Four themes were emerged specifically: 1) Knowledge of interprofessional learning and issue of horizontal learning, 2) Issues of dynamics in the learning process, 3) Challenges in implementing interprofessional learning, and 4) Suggestions for successful implementation of IPL.

## Knowledge of interprofessional learning and issue of horizontal learning

Our participants stated that they experience different levels of interprofessional learning throughout their medical years; namely during their undergraduate years and currently during their post-graduate study.

Our participants also stated that learning with other professionals generally helped them with knowledge gain, skill development, empowerment, and collaborative and communication skill.

They also acknowledged they have learned several skills from different levels of professionals including nurses, laboratory technicians, and pharmacists.

Another issue raised by our respondents was the importance of learning with other professionals in health care. They mentioned that it is beneficial for teaching and learning. Some of the benefits stated by our respondents were: improved quality of care, improved patient mismanagement, and help to stay updated. One of our respondents stated the following to describe this.

*…” From my experience professionals that are more knowledgeable and experienced work in government health institutions however due to the lack of smooth relationship between doctors and nurses’ quality of care is compromised, on the other hand, private health institutions are more successful at patient handling where Patients are more likely to get better, this is because of the teamwork, good working environment, and respect amongst professionals.”*

[Male, neurology R-3]

## Issues of power dynamics in the learning process

Our respondents have also indicated dynamics of interprofessional learning by acknowledging differences from the time they were undergraduate medical students, as general practitioners in their workplace, and now as residents at Tikur Anbessa specialized hospital.

In terms of communication with consultants, participants noted greater power differentials when they were undergraduate students, which improved significantly once they became residents.

Our respondents also mentioned that communication and learning change as the year of residency increases, where first-year residents usually learn informally and discuss mostly with second-year residents, not mostly with the consultant, however as the years progress communication occurs more with year senior residents and consultants.

## Challenges in implementing interprofessional learning

Respondents noted challenges related to the fact that Tikur Anbessa is a specialized, high-burden hospital. These include a lack of collaboration, varying codes of communication across and within departments, managerial and administrative issues, and preconceived bias and stereotypes. Our respondents commented:-

“……*Outside of the department, there is marginalization and biases. This is something I've noticed in our department as well.”* [Internal medicine R-3]

*“…….when the number of women increases, they believe it is because of affirmative action*”, *I faced this. When we first joined, they told us that the number of women had grown that year due to affirmative action. The guys then developed the mindset that they joined because they are qualified, but we are here because of affirmative action, and unless they double-check, no one wants to learn from women or follow orders, so we must work twice as hard and be twice as brilliant just to be regarded equal.” [Female, Internal medicine R3]*

## Suggestions for successful implementation of IPE

Our respondents noted gaps between, conceptualizing and stating goals for interprofessional learning, creating awareness, engaging students, having multidisciplinary academic sessions, and integrating interprofessional learning in the curriculum.

They nevertheless also indicated existing opportunities for interprofessional learning including the existence of many specialty departments at BLH, the presence of professional associations, the presence of different teaching methods, the presence of duty hour practice, and the presence of a virtual platform. One of our respondents stated the following to describe this.

*………… We learn a lot during our duty hours as we work with our colleagues in emergency and internal medicine. Because of different levels of professional assess patents, the first resident assesses the patient then another senior will evaluate and provide their detailed evaluation. As a result, we can learn a lot by reading the evaluation which will help us to understand the case better and see what we're missing.”* [Male, neurology R-3]

## Discussion

This study is one of the few studies [25] that analyzed the perceptions of readiness on interprofessional learning in post-graduate study in Ethiopia.

Analysis of qualitative data from neurology and internal medicine residents revealed that interprofessional learning is generally understood as a platform of learning within the same field and across different professions, which leads to collaborative work toward a common goal. This finding was partly reported on a systematic review [26]. Additionally, the RIPLS score of our study participants was high which strengthens the above statement, showing similar findings across both parts of our study. Similar finding was reported from United Arab Emirates [27]. The same finding was also reported from Iran [28] and Asia [20]. This evidence was also supported by a study done on medical students in Saudi Arabia [29–31]. This indicates that even though an interprofessional learning model has not yet been formally implemented, informal interprofessional learning is occurring among health professionals in this setting and the perceptions of readiness of the residents for the IPL is positive. This creates potential for fertile ground for the future incorporation of IPL into the curriculum.

In this study residents obtained the lowest mean score on sub-elements of RIPLS, for the statements ‘I would feel uncomfortable if another health care professional knew more about a topic than I did (2.42)’ and ‘There is little overlap between my role and that of other health care professionals(2.45)’. This finding is comparable with a study done in Malaysia with undergraduate students for the lowest mean score items [5]. The presence of a low mean score on the above sub-items suggests that the readiness of our residents in terms of professional identity is low, this needs further assessment at a broader level and work to increase their readiness level.

A study of paramedic students from Australia reported that students' readiness for Interprofessional learning did not appear to be significantly influenced by their gender; our study also revealed a similar finding [2]. However, a study done in the USA among healthcare students revealed more readiness for shared learning in female students [21] unlike our study. This discrepancy may be due to cultural differences.

Even though significant numbers of residents scored the lowest score on the professional identity subcategory of RIPLS, our participants reported that patient care and medication administration skills were primarily taught by nursing staff. Likewise, professionals like laboratory technicians, radiographers, and pharmacists are also mentioned as professions with whom inter-professional learning has taken place. This finding signifies the power of the qualitative portion of the study in capturing depicting the existing actual condition of interprofessional learning in our setup.

Most of our residents scored higher on the teamwork, collaboration and patient-centeredness subcategories of RIPLS. This finding also seen in our qualitative study. Our respondents mentioned interprofessional learning as having the following benefits: improvement in quality of care, improvement in patient mismanagement, and assistance with getting timely updates from a different discipline. The finding of the interviews also indicate that IPL learning is considered highly relevant to increase efficiency in time, money, and human resource by reducing unnecessary costs, reducing duplication of efforts, and avoiding long waiting time to receive care. All the above-mentioned situations may well have an impact on the quality of services and patient satisfaction. Similar findings were reported by a study done in Addis Ababa University on clinical psychology and psychiatry residents [25].

The existence of power dynamics within interprofessional learning was recognized by our respondents throughout their learning track by acknowledging differences from the time they were undergraduate medical students, as general practitioners in their workplace, and now as residents. A key point raised by our respondents was “first-year residents usually learn informally and discuss mostly with second-year residents, not mostly with the consultant”. This implies a possible communication gap between the first-year residents and the consultants, which needs further assessment and intervention to strengthen the interprofessional learning process.

Challenges raised by our respondents regarding interprofessional learning included**:** Tikur Anbessa specialized hospital being a high-burden hospital, lack of collaboration, varying codes of communication across and within the department for patient with a complicated case presentation, managerial and administrative issues, and preconceived bias and stereotypes. This finding was partly reported on in a study done in Addis Ababa University on clinical psychology and psychiatry residents [25]. Since the above-mentioned challenges significantly affect the interprofessional learning process it is important to consider these realities when implementing interprofessional learning in our setting. The identified presence of preconceived bias and stereotypes also requires consideration in planning new IPL activities.

Our respondents also made suggestions for successful implementation of IPL including recognizing that there is a gap, conceptualizing and stating the goals for interprofessional learning, creating awareness among the medical community, engaging students, engaging and integrating public health with specialties departments, integrating interprofessional learning in the curriculum, and making audit meetings multidisciplinary. Our survey participants pointed out several opportunities for interprofessional learning in our environment, such as the presence of multidisciplinary departments, professional associations, diverse teaching methods, and virtual platforms for mainstreaming. These resources will be essential for medical education experts at the institution to incorporate into our interprofessional learning initiatives.

Some limitations of this study are that it is an exploratory study of just two groups of residents at a single institution, and that only one professional group (medicine) was studied so there are no perspectives from other health professionals. Another limitation of this study is that very few studies have been done on IPL in postgraduate students. This limited our ability to compare our study findings with others. However, it is also a strength that this is one of the few [25] studies done on IPL in a postgraduate setting in Ethiopia.

In conclusion, we found a high score of RIPLS on internal medicine and neurology postgraduate residents, which implies readiness for interprofessional learning. Furthermore, the qualitative interviews indicated that neurology and internal medicine residents recognize interprofessional learning as a valuable learning platform. The challenges and opportunities identified in the study warrant further investigation for the future integration of interprofessional learning. It is noteworthy that medical residents' readiness for interprofessional learning does not seem to be significantly affected by factors such as age, gender, years of professional experience prior to postgraduate study, and department. This finding is promising for the future implementation of interprofessional learning programs and can guide medical education experts and policymakers in their decision-making.

### Supplementary Information

**Additional file 1.**

## Data Availability

The datasets used and analyzed during the current study are available from the corresponding author upon reasonable request.

## Abbreviations

IPL: Interprofessional learning
IPECP: Interprofessional Education and Collaborative Practice
IRB: Institutional Review Board
RIPLS: Readiness for Interprofessional Learning Scale
TASH: Tikur Anbessa Specialized Hospital
