# Supplementary material for: Perceptions of readiness for interprofessional learning among Ethiopian medical residents at Addis Ababa University: a mixed methods study
Source: BMC Med Educ. 2024 Jan 25;24:89. doi: 10.1186/s12909-024-05055-4 (PMC10811800; doi:10.1186/s12909-024-05055-4)
Supplement: Supplementary file 1 — Additional file 1. [file 12909_2024_5055_MOESM1_ESM.docx]

Supplementary material: Results of neurology and internal medicine residents' readiness for interprofessional learning scale score, TASH, from May 1 – June 30, 2021

| **Item No.** | **Statement** | **Strongly**  **Agree No ( %)** | **Agree No (%)** | **Undecided No (%)** | | **Disagree No (%)** | | **Strongly disagree No (%)** | |
| --- | --- | --- | --- | --- | --- | --- | --- | --- | --- |
| **Factor 1: Teamwork and collaboration** | | | | | | | | | |
| 9 | Shared learning will help me to think positively about other healthcare professionals | 74(73.3) | 25(24.8) | | 1(1) | | 0(0) | | 1(1) |
| 12 | Shared learning helps to clarify the nature of patient problems | 73(72.3) | 27(26.7) | | 0(0) | | 0(0) | | 1(1) |
| 10 | Shared learning with other healthcare professionals will help me to communicate better with patients and other professionals | 66(65.3) | 33(32.7) | | 1(1) | | 0(0) | | 1(1) |
| 13 | Shared learning before qualification would help healthcare professionals become better team workers | 67(66.3) | 31(30.7) | | 2(2) | | 0(0) | | 1(1) |
| 6 | Shared learning with other healthcare professionals will increase my ability to understand clinical problems | 60(59.4) | 35(34.7) | | 5(5) | | 1(1) | | 0(0) |
| 4 | Shared learning will help me understand my limitations | 59(58.4) | 36(35.6) | | 5(5) | | 0(0) | | 1(1) |
| 1 | Learning with other healthcare professionals will help me be a more effective member of a healthcare team | 60(59.4) | 35(34.7) | | 4(4) | | 2(2) | | 0(0) |
| 7 | Learning with health care students from other disciplines before qualification would improve relationships after qualification | 52(51.4) | 37(36.6) | | 10(9.9) | | 1(1) | | 1(1) |
| 8 | Communication skills should be learned with other healthcare professionals | 58(57.4) | 34(33.7) | | 7(6.9) | | 2(2) | | 0(0) |
| 11 | I would welcome the opportunity to work on small-group projects with other healthcare professionals | 52(51.5) | 38(37.6) | | 8(7.9) | | 2(2) | | 1(1) |
| 3 | Team-working skills are essential for all healthcare professionals to learn | 62(61.4) | 38(37.6) | | 0(0) | | 0(0) | | 1(1) |
| 2 | For small group learning to work, healthcare professionals need to trust and respect each other | 66(65.3) | 30(29.7) | | 4(4) | | 0(0) | | 1(1) |
| 5 | Patients ultimately benefit if healthcare professionals work together to solve patient problems | 84(83.2) | 16(15.8) | | 0(0) | | 0(0) | | 1 |
| **Factor 2: Patient centeredness** | | | | | | | | | |
| 26 | Establishing trust with my patients is important to me | 84(83.2) | 15(14.9) | 1(1) | | 0(0) | | 1(1) | |
| 29 | In my profession, one needs skills in interacting and cooperating with patients | 75(74.3) | 25(24.8) | 0(0) | | 1(1) | | 0(0) | |
| 28 | Thinking about the patient as a person is important in getting treatment right | 80(79.2) | 18(17.8) | 0(0) | | 2(1) | | 1(1) | |
| 25 | I like to understand the patient’s side of the problem | 74(73.3) | 25(24.8) | 0(0) | | 1(1) | | 1(1) | |
| 27 | I try to communicate compassion to my patients | 66(65.3) | 33(32.7) | 1(1) | | 0(0) | | 1(1) | |
| **Factor 3: Sense of professional identity** | | | | | | | | | |
| 17 | The function of nurses and therapists is mainly to provide support to doctors | 14(13.9) | 23(22.8) | 16(15.8) | | 30(29.7) | | 18(17.8) | |
| 16 | Clinical problem-solving skills should only be learned with professionals from my discipline | 13(12.9) | 13(12.9) | 11(10.9) | | 43(42.6) | | 21(20.8) | |
| 20 | I have to acquire much more knowledge and skills than other healthcare professionals | 25(24.8) | 37(36.6) | 15(14.9) | | 17(16.8) | | 7(6.9) | |
| 19 | I would feel uncomfortable if another healthcare professional knew more about a topic than I did | 8(7.9) | 17(16.8) | 13(12.9) | | 34(33.7) | | 29(28.7) | |
| 18 | There is little overlap between my role and that of other healthcare professionals | 8(7.9) | 15(14.9) | 12(11.9) | | 45(44.6) | | 21(20.8) | |
